# Inhibition of oxidative stress and advanced glycation end‐product formation in purified BSA/glucose glycation system by polyphenol extracts of selected nuts from Pakistan

**DOI:** 10.1002/fsn3.3331

**Published:** 2023-03-23

**Authors:** Asia Atta, Muhammad Shahid, Zunaira Kanwal, Saghir Ahmad Jafri, Muhammad Riaz, Hang Xiao, Mazhar Abbas, Chukwuebuka Egbuna, Jesus Simal‐Gandara

**Affiliations:** ^1^ Department of Biochemistry NUR International University Lahore 54000 Pakistan; ^2^ Department of Food Science University of Massachusetts Amherst 100 Holdsworth Way Amherst Massachusetts 01003 USA; ^3^ Department of Biochemistry University of Agriculture Faisalabad 38040 Pakistan; ^4^ Department of Biochemistry Allama Iqbal Medical College Lahore 54000 Pakistan; ^5^ Dean Faculty of Sciences NUR International University Lahore 54000 Pakistan; ^6^ Department of Allied Health Sciences University of Sargodha Sargodha 40100 Pakistan; ^7^ Department of Basic Sciences (Section Biochemistry) University of Veterinary and Animal Sciences Jhang Campus Lahore 35200 Pakistan; ^8^ Africa Centre of Excellence, Centre for Public Health and Toxicological Research (ACE‐PUTOR) University of Port‐Harcourt Port‐Harcourt Nigeria; ^9^ Nutrition and Bromatology Group, Analytical Chemistry and Food Science Department, Faculty of Science Universidade de Vigo E‐32004 Ourense Spain

**Keywords:** antiglycation, antioxidants, free radicals, hyperglycemia, polyphenols

## Abstract

Glycation generates advanced glycation end products (AGE) and its intermediates, thus increasing the risk of developing various ailments including diabetes mellitus. Current study was planned to explore the antioxidant and antiglycation potential of selected nuts viz, *Juglans regia* (Walnut), *Prunus dulcis* (Almond), *Pistacia vera* (Pistachio), and *Arachis hypogaea* (Peanut), locally available and readily consumed in Faisalabad, Pakistan, for their health‐promoting properties. The prepared methanolic extracts of selected nuts were tested for biological activities including the antioxidant and antiglycation potential. The effect of these extracts against oxidation and AGE formation was evaluated by in vitro method using bovine serum albumin (BSA)‐glucose system. *Juglans regia*, *Pistacia vera*, and *Arachis hypogaea* were found rich in phenolics and flavonoids contents with increased reducing potential and least IC_50_ due to the DPPH free radical scavenging inhibition. Dose‐ and time‐dependent inhibition of glucose‐induced advanced glycation end‐product (AGE) was exhibited by fruit extracts through in vitro bovine serum albumin (BSA)‐glucose system. *Juglans regia* and *Pistacia vera* were predominantly effective in the inhibition of early and intermediary glycation products at different incubation conditions. The study indicated that the extracts of selected nuts possess significant antioxidant capacity and are rich in phenolics and flavonoids, making them useful supplements as an important part of a balanced diet.

## Graphical abstract

All the studied nuts have high phytonutrients content with significant antioxidant and antiglycative properties. At a dosage of 5 mg/mL, all the studied extracts successfully prevent glucose‐mediated protein modification in vitro during the 18–24th days of incubation. Plant‐derived foods loaded with bioactive moieties are beneficial in the inhibition of oxidative stress and AGEs formation in customizing a healthy diet for the target group.

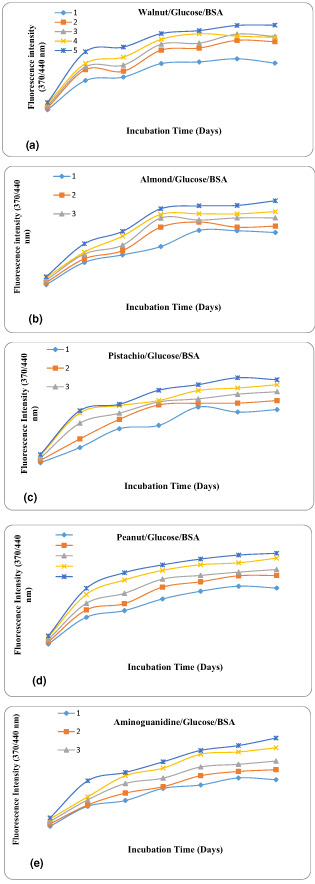

## 1 INTRODUCTION

Dietary guidelines that emphasize on the dynamic characteristics of phytonutrients have valuable impact on human health against a variety of diseases (Shahidi, 2009; Zia‐Ul‐Haq et al., 2014). The consumption of some fruits and vegetables is inversely correlated with disease morbidity, which may be due to their antioxidant potential (Wootton‐Beard & Ryan, 2011). However, it is widely agreed that some synthetic antioxidants such as butylhydroxyanisole and butylhydroxytoluene should be replaced with natural antioxidants due to their suspected toxicity and associated health hazards (Zhang & Liu, 2015).

Age‐related nonenzymatic production and accumulation of AGEs (advanced glycation end products) have been linked to the increased progression of diabetes as well as the pathogenesis of a variety of other disorders including neurodegeneration and diabetic vascular complications (Lee et al., 2007). Hyperglycemia in diabetes induces oxidative stress which increases free radical production owing to glucose auto‐oxidation and disturbs the electron transport chain (Ahmad et al., 2009; Mustafa et al., 2019). In humans, oxidative stress is caused by the disruption of natural antioxidant defense systems, usually consisting of reactive oxygen species (ROS) which is linked to a variety of diseases (Fatima et al., 2022; Riaz et al., 2017). Dietary phytonutrient supplementation offers a variety of defense mechanisms against different ailments, resulting in improved general health. Some developing countries like Pakistan are rich in fruits and vegetables but have not yet thoroughly tested for their health‐promoting properties. The current study was planned to explore the antioxidant and anti‐glycation potential of widely used and easily available nuts as dietary supplements.

## 2 MATERIALS AND METHODS

### 2.1 Sample collection and identification

The selected nuts, that is, *Juglans regia*, *Prunus dulcis*, *Pistacia vera*, and *Arachis hypogaea*, used for the current study were obtained from the local market of Faisalabad, Pakistan. These nuts materials were identified and authenticated from Department of Botany, University of Agriculture Faisalabad, Pakistan, and grinded to fine powder, mixed thoroughly, and stored at −20°C for further analysis.

### 2.2 Extraction of crude polyphenols

The crude polyphenols from selected nuts materials were extracted following the reported method of Chandrasekara and Shahidi (2011) with slight modification. Nuts materials (25 g each nut separately) were blended in 100 mL of chilled 80% ethanol in 1:8 w/v for 10 min with the help of a warming blender and then homogenized the mixture for 5 min in a Virtis High Speed Homogenizer followed by filtration under vacuum. The mixture was then washed using n‐hexane (50 mL) through centrifugation at 2500 *g* for 10 min. Following the removal of n‐hexane fraction, the extract was washed twice and the ethanol/water mixture was evaporated to dryness under vacuum at 45°C using rotary evaporator. The extracts were stored at −40°C until further analysis.

### 2.3 Antioxidant activity of crude polyphenol extracts

#### 2.3.1 Total phenolic contents (TPC)

Standard Folin–Ciocalteu method was used for the determination of TPC using gallic acid as the standard. Briefly, equal volume of extract (500 μL; 5 mg of extract dissolved in 5 mL of 1:1 v/v methanol/water) was mixed with Folin–Ciocalteu reagent (500 μL) and kept for 5 min at room temperature. Following the addition of 20% Na_2_CO_3_ (1.0 mL), the mixture was incubated at room temperature for 10 min and centrifuged at 150 *g* for 8 min. Then, 200 μL supernatant was transferred to micro‐well plate followed by measuring the absorbance of each well at 730 nm. The total phenolic content was expressed as gallic acid equivalents (GAE) in mg/g of sample (Noor et al., 2014).

#### 2.3.2 Total flavonoids contents (TFC)

For the determination of TFC, 1 mL extract of each selected nut (10 mg/mL) was placed in a 10‐mL volumetric flask followed by the addition of 5 mL distilled water and 0.3 mL of 5% NaNo_2_. After 5 min, 0.6 mL of 10% AlCl_3_ was added to the mixture followed by the addition of 2 mL of 1 M NaoH. Absorbance of the mixture was measured at 510 nm photometrically and the amount of TFC was expressed as mgCE/g dry weight of the sample (Barros et al., 2011).

#### 2.3.3 DPPH radical scavenging assay

The ability of extract to scavenge 2,2‐diphenyl‐1‐1‐picrylhydrazyl stable radicals was used to measure the antioxidant potential (Muhammad Riaz et al., 2012); 50 μL aliquot of different sample concentrations was mixed with 5 mL of 0.004% DPPH solution. Following 30 min incubation at room temperature, absorbance of the mixture was measured photometrically at 517 nm against reagent blank.

$$\%I=\left(A\;\text{blank}–A\;\text{sample}/A\;\text{blank}\right)\times 100$$

Where %*I*: percent inhibition, A blank: absorbance of blank, A sample: absorbance of sample.

#### 2.3.4 Determination of reducing power

The reducing power of the extracts was determined as described by Barros et al. (2011). Briefly, 5 mL of sample was mixed with 5 mL of 0.2 M phosphate buffer (pH: 6.6) followed by the addition of 5 mL of 1% potassium ferricyanide. After 20 min of incubation at 50°C, 5 mL of 10% trichloroacetic acid was added to the mixture and then centrifuged at 12,000 *g* for 10 min. The upper layer of the solution (2.5 mL) was mixed with distilled water (5.0 mL) and 0.1% ferric chloride solution (0.5 mL). The absorbance of the mixture was measured at 700 nm.

### 2.4 Antiglycation activity of crude polyphenol extracts

#### 2.4.1 Nonenzymatic protein glycation

For the determination of nonenzymatic protein glycation, 10 mL of 20 mg/mL bovine serum albumin (BSA) was mixed with 5 mL of 500 mM glucose and 0.02% sodium azide in 200 mM phosphate buffer (pH 7.4). Different concentrations of sample dissolved in 5 mL of 200 mM phosphate buffer (pH 7.4) was mixed with the reaction mixture followed by incubation at 37°C for 30 days to obtain the glycated materials using aminoguanidine as the positive control (Wu et al., 2009).

#### 2.4.2 Nitro‐blue tetrazolium (NBT) reductive assay

For NBT reductive assay, 0.5 mL of glycated material and 2 mL of 0.3 mM NBT reagent in 100 mM Na_2_CO_3_ were incubated at room temperature for 15 min. Absorbance of the mixture was measured at 530 nm against a blank (Zhang et al., 2011).

#### 2.4.3 Girard‐T assay

Girard‐T assay was used for the photometric analysis of dicarbonyl compounds. Briefly, 0.4 mL of glycated material was incubated with 0.2 mL of 500 mM Girard‐T stock solution and 3.4 mL of 500 mM sodium format (pH 2.9) for 1 hour at 37°C. The absorbance of the mixture against the blank was measured at 294 nm with the help of UV‐2550 spectrophotometer (Shimadzu). The blank solution contains all the reagents except Girard‐T stock solution (Li et al., 2014; Zhang et al., 2011).

#### 2.4.4 AGEs analysis

The glycated material (0.5 mL) was diluted with distilled water making the volume up to 10 mL. The fluorescence of the sample was measured at 370 and 440 nm for excitation and emission, respectively, using an F‐5301 spectrofluorometer (Shimadzu; Zhang et al., 2011).

### 2.5 Statistical analysis

Mean ± SD of replicate measurements were computed using Microsoft Excel 7.0. One‐way analysis of variance (ANOVA) followed by multiple comparison test was applied to determine the statistically significant differences of tested biological activities for the studied nuts extract using Minitab 13.1 statistical software. *p* < .05 was considered statistically significant.

## 3 RESULTS AND DISCUSSION

### 3.1 Antioxidant potential of polyphenolic extracts of nuts

Antioxidants are the agents or chemicals that have the free radical scavenging potential and prevent the production of ROS. Medicinal plants, herbs, and vegetables are the natural sources of variety of substances called bioactives, which have many biological activities including antioxidant potential (Kauser et al., 2018). Recently, the use of natural antioxidants in food industry and health care has gained much importance. A large number of phytobioactive compounds such as phenolics and flavonoids are found in nonedible and edible plants including nuts and are associated with various bio‐pharmacological activities (Shahid et al., 2022). Because of the significant role of natural antioxidants in improving the general health, we in the current research evaluated the antioxidant potential of selected nuts extract.

#### 3.1.1 Total phenolics contents (TPC) and total flavonoids contents (TFC)

Nuts are high in several therapeutically active constituents, particularly polyphenols (Carvalho et al., 2010). The results of TPC and TFC of studied nuts are given in Table 1. All studied nuts extracts possess significant amounts of total phenolics ranging from 1.68 to 8.44 mg GAE/g, and total flavonoids ranged from 0.73 to 3.78 mg CE/g. The highest phenolics (8.44 mg GAE/g) and flavonoids (3.78 mg CE/g) contents were found in *Juglans regia*, while the least phenolic contents (1.68 ± 0.01 mg GAE/g) and flavonoids contents (0.73 ± 0.02 mg CE/g) were found in *Prunus dulcis*. The tannin content of walnuts and almonds was previously reported in published study of Amarowicz and Pegg (2008). *Juglans regia* (Walnut) methanolic extracts have the highest total flavonoids contents, which agrees with various other research studies that demonstrated *J. regia* seeds to be a rich dietary source of phenolics (Chen & Blumberg, 2008; Yang et al., 2009). However, seasonal, agronomic practice variables, genomics, moisture levels, extraction method, and the standard used, among some other factors, could explain the discrepancies in polyphenolic content of fruit (Kalpna & Mital, 2011).

**TABLE 1:** Antioxidant potential of polyphenolic extracts of selected nuts.

Sr. No	Scientific name	TPC(mg GAE/g)	TFC(mg CE/g)	DPPH(IC_50_ mg/g)	R. Power
1	*Juglans regia*	8.44 ± 0.02^c^	3.78 ± 0.21^c^	0.980 ± 0.48^a^	2.21 ± 0.06^c^
2	*Prunus dulcis*	1.68 ± 0.01^a^	0.73 ± 0.70^a^	4.254 ± 0.05^d^	1.03 ± 0.04^a^
3	*Pistacia vera*	6.9 ± 0.05^b^	2.32 ± 0.50^ab^	1.548 ± 0.04^b^	1.98 ± 0.11^b^
4	*Arachis hypogaea*	3.06 ± 0.01^a^	1.80 ± 0.23^ab^	2.538 ± 0.01^c^	1.43 ± 0.06^a^

*Note*: Values are mean ± SD of carefully conducted triplicate experiments. Superscripted alphabets within a column indicate significance (*p* < .05) observed at 95% confidence interval (CI).

#### 3.1.2 Reducing power assay of selected nuts

The reducing potential of the studied dry fruits extracts is increased with increasing concentration. Present study was planned to compare the reducing potential of the methanolic extract of studied nuts with BHA and the results are given in Table 1. Dose–response investigations revealed a positive linear relationship between the concentration of nut extracts and the reducing power prior to reaching the threshold level. All the studied nuts extracts give absorbance values above 0.65 except for the *J. regia* extract which gives absorbance value of 2.21. Significant similarities were observed between almond and peanut extracts with the lowest reducing potential of almond. According to the published reports, the reducing potential is associated with the development of reductones, acting as antioxidants to disrupt the free radical chain by donating a hydrogen atom (Nisha et al., 2009; Ribeiro et al., 2008). These findings of our study are in agreement with the findings of published study which reported the highest reducing potential of *P. dulcis* and *J. regia* (Mishra et al., 2010).

#### 3.1.3 DPPH radical scavenging activity of nuts

The antioxidant activity results of dry fruits extract are given in Table 1. Methanolic extract of almond showed lowest radical scavenging potential, while the extract of walnut and pistachio were found to be the most potent DPPH radical scavengers, with IC50 values of 0.98 ± 0.48 mg of extract/g of dry weight and 1.55 ± 0.04, respectively. This can be attributed to their higher total phenolic content when compared to certain other fruits. The selected fruits were ranked according to their scavenging capacity, based on absolute numbers rather than statistical differences as >pistachio >walnut >peanut >almond. Deve et al. (2014) reported that phenolics and flavonoids in nuts and peanuts account for a substantial portion of total antioxidant activity, implying that a combined effect of phytochemical and synergistic mechanisms in the nut matrix may be able to take responsibility for their potent antioxidant activities.

### 3.2 Antiglycation potential of polyphenolic extracts of selected nuts

The Maillard process is a complex set of reactions that involves in reducing the sugars and proteins and eventually giving rise to AGEs. The events that contribute to the breakdown of proteins produced by glucose can be classified into three types: (i) those that overlap and produce early Amadori products, (ii) those that are intermediate (cross linking of protein and creation of carbonyl groups), and (iii) those that are final and make post‐Amadori adducts (Miroliaei et al., 2011). These AGEs have the potential to aggravate diabetic issues as well as other neurodegenerative conditions such as Alzheimer's disease. As a result, AGE inhibitors may have therapeutic properties against various disorders. AGE inhibitors naturally derived from foods have gained popularity, and the basic concept appears to form the basis for the reduction of glycol‐oxidation, reactive 1,2‐dicarbonyl compound oxidative stress, reactive nitrogen, and oxygen species (Ho et al., 2010). Indeed, numerous dietary plants and their constituents have better anti‐glycation properties to aminoguanidine (Peng et al., 2008; Spagnuolo et al., 2021).

#### 3.2.1 Effect of selected nuts extract on the reduction of NBT

The NBT reduction test was used to determine the inhibitory potential of studied nuts extract on the production of an early‐stage glycation product (Amadori product). The results showed that all the edible components exhibited higher inhibitory effects in a dose‐dependent manner, with the peak inhibitory activity occurring on the eighteenth day of incubation at a dose of 5 mg/mL. Walnut (46%), pistachio (43%), and peanut (43%), as compared to aminoguanidine (38%), were found to have considerable inhibitory activity on the reduction of NBT at 5 mg/mL. Almond, on the other hand, was 35% as efficient as aminoguanidine in reducing the inhibition. The ability of tested samples to inhibit NBT reduction was in the following sequence: walnut (19%–46%) > pistachio (24%–43%) > peanut (19%–43%) > aminoguanidine (10%–38%) > almond (11%–35%) (Table 2).

**TABLE 2:** Inhibitory effect of polyphenolic extracts of different nuts on the reduction of NBT induced by glycated/BSA system.

Scientific name	Inhibition (%)				
	Concentrations of polyphenolic extracts (mg/mL)				
	1	2	3	4	5
*Juglans regia*	19.88 ± 0.04_a_^c^	25.61 ± 0.04_b_^b^	35.44 ± 0.00_c_^bc^	41.25 ± 0.01_cd_^d^	46.45 ± 0.03_d_^d^
*Prunus dulcis*	11.82 ± 0.09_a_^b^	15.30 ± 0.01_a_^a^	25.17 ± 0.02_b_^a^	30.45 ± 0.07_c_^a^	35.55 ± 0.01_d_^a^
*Pistacia vera*	24.02 ± 0.01_a_^c^	27.74 ± 0.02_a_^b^	36.52 ± 0.01_b_^c^	39.45 ± 0.01_c_^c d^	43.16 ± 0.00_d_^c^
*Arachis hypogaea*	19.16 ± 0.00_a_^c^	26.32 ± 0.02_b_^b^	37.63 ± 0.02_c_^c^	41.61 ± 0.02_d_^d^	43.36 ± 0.02_d_^c^
Aminoguanidine	10.79 ± 0.00_a_^a^	21.61 ± 0.00_b_^b^	30.48 ± 0.00_c_^c^	37.11 ± 0.00_d_^c^	38.89 ± 0.00_d_^b^

*Note*: BSA (20 mg/mL) was incubated with glucose (500 mm) in phosphate buffer (0.2 M, pH 7.4) at 37°C for 18 days. Values are mean ± SD of carefully conducted triplicate experiments. Superscripted and subscripted alphabets within a column and row indicate significance (*p* < .05) observed at 95% confidence interval (CI) between plant investigated and concentration applied, respectively. 1, 2, 3, 4, 5 (mg/mL) are the concentrations of polyphenolic extracts. Aminoguanidine is used as positive control.

Because albumin and other proteins have a half‐life of 2 weeks, and measuring early‐stage glycation product (Miroliaei et al., 2011) seems to be the method utilized to quantify protein AGE adducts (Jeppsson et al., 2002; Lapolla et al., 2005). The capacity of ketoamine family to reduce the dye nitro blue tetrazolium (NBT) and synthesize a molecule that absorbs at 525 nm is used by scientists to detect fructosamine (Lapolla et al., 2005). The lower absorbance of the treated samples of various plant extracts suggests their efficiency in reducing glycation (Zhang et al., 2011). A considerable reduction in glycation reaction (percent inhibition) demonstrates the extract's potential to behave as a natural inhibitor attenuating Millard response at the early stage (Matsuura et al., 2002).

#### 3.2.2 Effect of selected nuts on the formation of α‐dicarbonyls

Published studies reported that dicarbonyl chemicals (glyoxal, methyl glyoxal, and deoxyglucosones) accelerate the protein cross‐linking and formation of stable AGEs. According to a study, dicarbonyl chemicals account for 45%–50% of AGEs (Ruggiero‐Lopez et al., 1999). We, in the current study, found that the quantity of dicarbonyl compounds is increased during first 18 days of incubating the dried fruit extracts with the glucose/ BSA model. All the studied nuts extract and aminoguanidine at a concentration of 5 mg/mL inhibited the synthesis of dicarbonyl compounds on the eighteenth day of incubation. Table 3 reveals the dose‐dependent inhibitory effects of several dry fruit methanolic extracts on dicarbonyl compound formation. All the studied nuts except peanuts inhibited the BSA glycation systems more than aminoguanidine at 5 mg/mL dose (38.90%). Pistachio at a dose of 1 mg/mL showed the highest inhibitory activity on the formation of dicarbonyl chemicals in BSA glycation processes. All edible commodities, including pistachio, walnut, almond, aminoguanidine, and peanuts, had inhibitory effects ranging from 23% to 51%, 24% to 48%, 15% to 46%, 12% to 40%, 13% to 40%, and 15% to 38%, respectively.

**TABLE 3:** Inhibitory effect (%) of polyphenolic extracts of different dry fruits on the formation of α‐dicarbonyl compounds in glucose/BSA system.

Scientific name	Inhibition (%)/concentration (mg/mL)				
	1	2	3	4	5
*Juglans regia*	15.29 ± 0.01^b^_a_	19.52 ± 0.01^a^_b_	34.95 ± 0.01^bc^_c_	42.38 ± 0.01^d^_d_	46.26 ± 0.01^c^_d_
*Prunus dulcis*	12.27 ± 0.01^a^_a_	25.92 ± 0.01^b^_b_	35.52 ± 0.00^c^_c_	38.85 ± 0.01^c^_c_	40.86 ± 0.00^b^_d_
*Pistacia vera*	24.66 ± 0.00^c^_a_	27.98 ± 0.01^c^_a_	38.91 ± 0.01^c^_c_	45.28 ± 0.01^d^_d_	48.49 ± 0.01^cd^_d_
*Arachis hypogaea*	15.15 ± 0.01^b^_a_	26.42 ± 0.01^b^_b_	30.02 ± 0.01^b^_c_	32.57 ± 0.00^b^_c_	38.90 ± 0.01^a^_d_
Aminoguanidine	13.26 ± 0.01^b^_a_	19.47 ± 0.00^a^_b_	26.88 ± 0.01^a^_c_	30.40 ± 0.01^a^_c_	40.77 ± 0.01^b^_d_

*Note*: BSA (20 mg/mL) was incubated with glucose (500 mm) in phosphate buffer (0.2 M, pH 7.4) at 37°C for 18 days. (means ± SD, *n* = 3). Superscripted and subscripted alphabets within a column and row indicate significance (*p* < .05) observed at 95% confidence interval (CI) between plant investigated and concentration applied, respectively. 1, 2, 3, 4, 5 (mg/mL) are the concentrations of polyphenolic extracts. Aminoguanidine is used as positive control.

#### 3.2.3 Effect of selected dry fruits extract on the formation of AGEs

**FIGURE 1:**
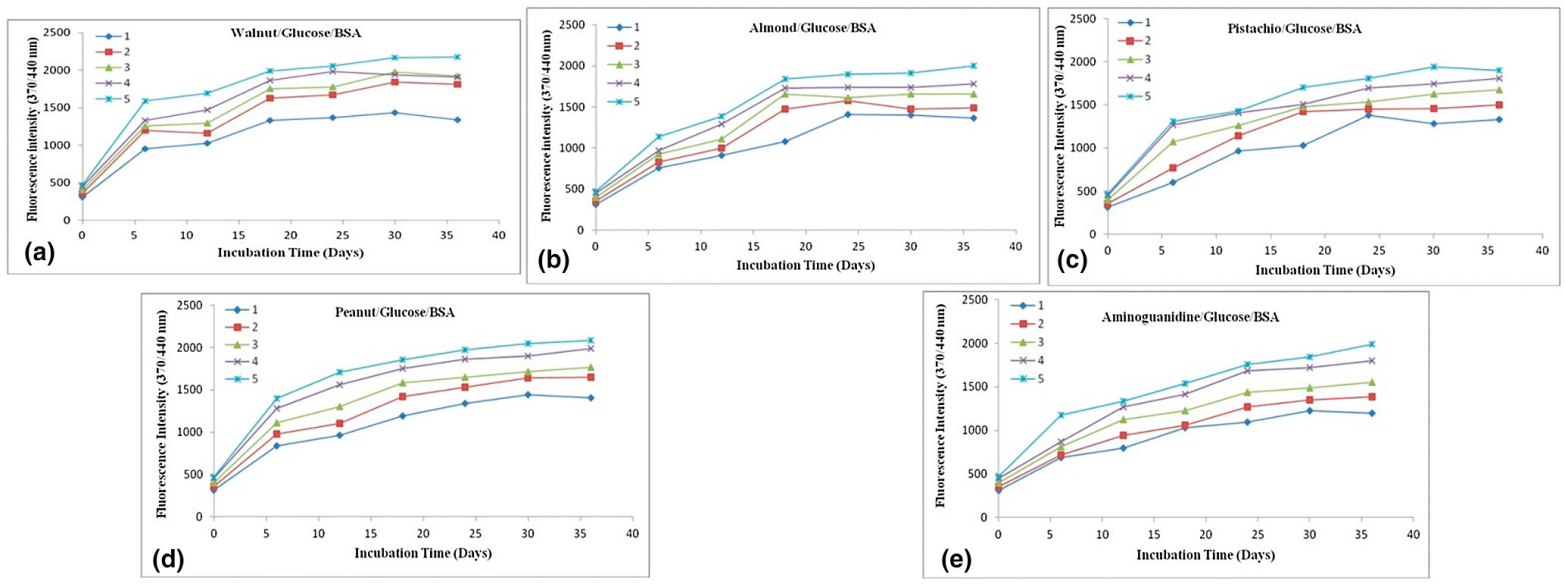
(a–e) Effect of selected dry fruits and aminoguanidine at different concentrations (1–5 mg/mL) on fluorescent AGEs formation in BSA incubated with glucose (1 M) at 0–36 days. (a) BSA incubated with glucose and Walnut (1, 2, 3, 4, 5 mg/mL) (b) BSA incubated with glucose (1 M) and Almond (1, 2, 3, 4, 5 mg/mL), (c) BSA incubated with glucose (1 M) and Pistachio (1, 2, 3, 4, 5 mg/mL), (d) BSA incubated with glucose (1 M) and Peanut (1, 2, 3, 4, 5 mg/mL), (e) BSA incubated with glucose (1 M) and Aminoguanidine (1, 2, 3, 4, 5 mg/mL). All the graphs are plotted from the mean values of replicate measurements.

In vitro inhibitory potential of methanolic extract of dry fruits on the formation of AGEs was assessed by determining the fluorescence emission using aminoguanidine as standard AGEs inhibitor. All the studied extracts inhibited the formation of AGEs in BSA glycation systems (Table 4). Fluorescence intensity of albumin in various glycation systems of selected dry fruits at different concentrations is plotted in Figure 1 showing the effect of selected dry fruits and aminoguanidine at different concentrations (1–5 mg/mL) on fluorescent AGEs formation in BSA incubated with glucose (1 M) at different incubation periods; (A) BSA incubated with glucose (1 M) and Walnut (1, 2, 3, 4, 5 mg/mL), (B) BSA incubated with glucose (1 M) and Almond (1, 2, 3, 4, 5 mg/mL), (C) BSA incubated with glucose (1 M) and Pistachio (1, 2, 3, 4, 5 mg/mL), (D) BSA incubated with glucose (1 M) and Peanut (1, 2, 3, 4, 5 mg/mL), and (E) BSA incubated with glucose (1 M) and Aminoguanidine (1, 2, 3, 4, 5 mg/mL). All the graphs are plotted from the mean values of replicate measurements. The fluorescence of the BSA/glucose system rises by several orders of magnitude after 24 days of incubation at 37°C (Figure 1). However, the fluorescence produced by AGE was inhibited clearly when sample extracts and aminoguanidine were introduced to the incubating system. Walnut (49.46%) and pistachio (47.89%), in particular, have higher antiglycative effects than aminoguanidine (45.83%). In the glycated BSA model, all the studied nuts except pistachio showed their maximum inhibitory antiglycative activities at eighteenth day of incubation (not indicated in the Table 4). At 1 mg/mL, walnut and peanuts had the highest inhibitory effects, followed by almond at 4 mg/mL and pistachio at 5 mg/mL (Figure 1). The ability of the studied nuts extract to function as natural inhibitors attenuating Millard reaction is demonstrated by a significant decrease in glycation reaction at low doses, notably in the case of walnut and peanuts (Matsuura et al., 2002).

**TABLE 4:** Inhibitory effect of polyphenolic extracts of different dry fruits on the formation of AGEs in glucose/BSA system.

Scientific name	Inhibition (%)/concentration (mg/mL)				
	1	2	3	4	5
*Juglans regia*	**49.46 ± 1.93** ^**d**^_**d**_	32.46 ± 1.47^c^_d_	11.48 ± 1.47^a^_a_	21.39 ± 2.64^b^_b_	27.70 ± 0.96^c^_c_
*Prunus dulcis*	10.95 ± 0.89^a^_b_	18.71 ± 1.45^b^_b_	27.83 ± 1.80^c^_c_	**46.23 ± 1.54** ^**d**^_**d**_	27.23 ± 1.59^c^_c_
*Pistacia vera*	31.36 ± 1.70^a^_c_	39.19 ± 1.15^b^_c_	38.62 ± 1.42^b^_d_	42.81 ± 1.33^d^_d_	**44.89 ± 1.58** ^**d**^_**d**_
*Arachis hypogaea*	**36.14 ± 1.38** ^**d**^_**c**_	23.20 ± 1.43^c^_b_	20.40 ± 1.19^b^_b_	15.11 ± 1.03^a^_a_	22.62 ± 1.86^b^_c_
Aminoguanidine	23.55 ± 0.62^a^_d_	27.97 ± 1.18^a^_c_	37.59 ± 0.89^b^_c_	41.55 ± 1.09^c^_c_	**44.57 ± 0.56** ^**d**^_**d**_

*Note*: At 30°C, glucose (500 mM) was treated in phosphate buffer (0.2 M, pH 7.4) both with and without BSA (20 mg/mL) for 24 d (mean ± SD, *n* = 3). Superscripted and subscripted alphabets within a column and row indicate significant (*p* < .05) observed at 95% confidence interval (CI) between plant investigated and incubation time at concentration applied, respectively. 1, 2, 3, 4, 5 (mg/mL) are the concentrations of polyphenolic extracts. Aminoguanidine is used as positive control.

Bold values represent the highest inhibitory concentration.

Thus, according to Lunceford and Gugliucci (2005), the decrease in AGE production in natural substances was mostly inhibiting the activity of the second stage of glycation mechanisms, such as the free‐radical induced conversion of Amadori products to AGE. Despite the reality that no oxidation process is believed to be involved in the formation of Amadori recombination products, it is hypothesized that oxidation is linked to the generation of fluorescence and molecular crosslinking, both of which are AGE characteristics (Fu et al., 1994). Several investigations have indicated that plants extracts including ginseng (Bae & Lee, 2004), Luobuma tea (Yokozawa & Nakagawa, 2004), Finger millet (*Eleusine coracana*), and Kodo millet (*Paspalum scrobiculatum*) (Hegde et al., 2002) inhibit the formation of glycated proteins. Therefore, the inhibiting effect of selected dry fruits extract on protein oxidation induced by glycation may be because of their antioxidant activities. Moreover, biologically active constituents of selected dry fruits remain unknown. In order to prove this hypothesis, HPLC‐MS analysis is required for the separation and characterization of bioactive compounds from the selected dry fruits.

## 4 CONCLUSION

The current study concluded that all the studied nuts particularly *J. regia* and *P. vera* have high phytochemical contents with significant antioxidant and antiglycative properties. At a dose of 5 mg/mL, all the studied nuts extract successfully inhibited the glucose‐mediated protein modification in vitro during the 18–24th days of incubation, depending on the sample type. Plant‐derived foods loaded with bioactive moieties are beneficial in this situation in customizing a healthy diet for the target group. In order to increase our understanding of biological potential, further in vivo research is required. The modes of action as well as the structural elucidation of these fruits' potent antiglycative chemicals and the nutraceutical value of these examined consumables in mind; this research opens up new avenues towards the use of functional/nutraceutical foods and their bioactive moieties as therapeutic agents.

## AUTHOR CONTRIBUTIONS

**Asia Atta:** Conceptualization (equal); data curation (lead); formal analysis (lead); investigation (lead); methodology (lead); writing – original draft (lead). **Muhammad Shahid:** Conceptualization (equal); funding acquisition (equal); project administration (lead); resources (lead); supervision (lead); validation (equal); writing – review and editing (equal). **Zunaira Kanwal:** Data curation (supporting); formal analysis (equal); methodology (supporting); visualization (supporting); writing – review and editing (equal). **Saghir Ahmad Jafri:** Conceptualization (supporting); investigation (supporting); validation (equal); visualization (supporting); writing – review and editing (equal). **Muhammad Riaz:** Formal analysis (equal); investigation (equal); methodology (supporting); validation (supporting); writing – review and editing (lead). **Hang Xiao:** Conceptualization (supporting); formal analysis (supporting); funding acquisition (equal); methodology (equal); supervision (equal); validation (equal); writing – review and editing (supporting). **Mazhar Abbas:** Data curation (equal); methodology (equal); resources (supporting); software (supporting); writing – review and editing (supporting). **Chukwuebuka Egbuna:** Investigation (equal); supervision (supporting); visualization (equal); writing – review and editing (supporting). **Jesus Simal‐Gandara:** Funding acquisition (equal); validation (equal); visualization (equal); writing – review and editing (equal).

## FUNDING INFORMATION

Higher Education Commission (HEC), Government of Pakistan provided the financial support under the indigenous PhD program.

## CONFLICT OF INTEREST STATEMENT

Authors declare that they have no conflict of interest.

## CONSENT FOR PUBLICATION

Not applicable.

## ETHICAL APPROVAL

The research work was approved by the research scrutiny committee of the University of Agriculture, Faisalabad, Pakistan.

## Data Availability

Data are available from the authors upon reasonable request.
